# NCAPG stimulates lung adenocarcinoma cell stemness through aerobic glycolysis

**DOI:** 10.1111/crj.13676

**Published:** 2023-08-08

**Authors:** Zuwang Zhang, Dongdong Qi, Xun Liu, Poming Kang

**Affiliations:** ^1^ Department of Thoracic Surgery University‐Town Hospital of Chongqing Medical University Chongqing China

**Keywords:** cell stemness, glycolysis, lung adenocarcinoma, NCAPG

## Abstract

**Background:**

Cancer stem cells are pivotal in cancer progression and therapy, including lung adenocarcinoma (LUAD). High NCAPG level is implicated in malignant tumorigenesis, but investigations on NCAPG and LUAD stem cells are warranted. Hence, projecting the impact of NCAPG on cell stemness and the targeted therapy for LUAD is of the essence.

**Methods:**

Bioinformatics analyzed NCAPG expression in LUAD tissues. qRT‐PCR assayed NCAPG expression in LUAD cells. CCK‐8 assessed cell viability and cell sphere‐forming assay measured sphere‐forming ability. Western blot assessed expression of stem cell‐related markers (CD133, CD44, Oct‐4) and specific genes (HK2, PKM2, LDHA) related to glycolysis metabolism pathway. Cellular glycolytic capacity was assayed by glycolytic metabolites pyruvic acid, lactate, citrate, and malate assay kits, and extracellular acidification rate and oxygen consumption rate analyzers.

**Results:**

NCAPG was upregulated in LUAD and enriched in the aerobic glycolysis pathway, and its expression was positively correlated with that of glycolytic marker genes. Cell function assays revealed that NCAPG stimulated proliferation, stemness, and glycolytic activity of LUAD cells. Rescue experiments unveiled that 2‐DG (glycolysis inhibitor) was able to reverse the stimulative impact of NCAPG overexpression on proliferation, stemness, and glycolytic activity of LUAD cells.

**Conclusion:**

NCAPG stimulated LUAD cell stemness through activation of glycolysis pathway. NCAPG may be possible biomarker for diagnosis and target for treatment of LUAD.

## Graphical abstract

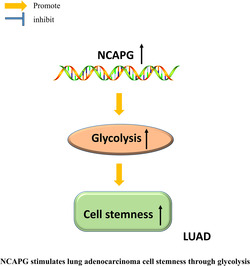

## 1 INTRODUCTION

Lung adenocarcinoma (LUAD) is a malignant and fatal respiratory disease with high mortality.^1^ It is hard to be diagnosed in the early stage, and most patients preclude surgery with diagnosis in the advanced stage.^2^ Clinical outcomes for LUAD patients have been improved remarkably with advances in diagnosis, surgery, radiotherapy, and molecular therapy, but the 5‐year survival is dissatisfying.^3^ Previously, molecular mechanisms affecting LUAD have been probed. Liang et al^4^ reported that circDCUN1D4 constrains metastasis of LUAD in a TXNIP‐dependent manner. Thence, underlying molecular mechanism modulating LUAD is of concern for therapy.

Cancer stem cells (CSCs) are a population of self‐renewing cells with high tumorigenic capacity that can readily adapt to changes in the environment and are more resistant to conventional therapies than other cells, thus playing a critical role in tumorigenesis, recurrence, and metastasis.^5^, ^6^ Chen et al^7^ revealed that sodium selenite is able to attenuate LUAD progression via repressing SOX2‐mediated stemness. Liu et al^8^ unveiled a positive feedback loop between high levels of RFC4 and NICD1 and sustained over‐activation of the Notch signaling, thereby facilitating metastasis and stemness in non‐small cell lung cancer. Ganguly et al^9^ unraveled that MUC5AC stimulates pancreatic cancer progression through cell stemness by upregulating Klf4 through phosphorylated STAT3. Additionally, tumor cell glycolysis is closely related to CSCs. Zhu et al^10^ unraveled that ETV4 enhances glycolytic activity and stemness in breast cancer. A growing body of evidence reveals mechanisms that affect cancer cell stemness, but the molecular mechanisms involved in LUAD cell stemness are underexplored. Thus, this study attempted to dig out underlying factors that modulated LUAD cell stemness.

The aberrant expression of non‐SMC condensin I complex subunit G (NCAPG), a subunit of condensin 1, facilitates tumor progression.^11^ Tang et al^12^ illustrated that NCAPG is substantially upregulated in bladder cancer and fosters cell proliferation through NF‐kappaB signaling pathway. Gong et al^13^ manifested that NCAPG is noticeably upregulated in hepatocellular carcinoma tissues and cells and exerts an oncogenic role in cell proliferation and antiapoptosis through activation of PI3K/AKT/FOXO4 pathway. Wang et al^14^ reported that NCAPG is significantly prominently upregulated in LUAD and stimulates proliferation and migration by elevating p‐Smad2 and p‐Smad3 expression in TGF‐β signaling pathway. The mechanism of NCAPG in cancer cell stemness is not yet clear, and thus clarifying molecular mechanism of NCAPG in LUAD progression is indispensable for therapy.

In this study, we analyzed the expression and cellular function of NCAPG in LUAD to clarify the mechanism of NCAPG influencing cell stemness in LUAD. NCAPG was markedly upregulated in LUAD tissues and cells, and NCAPG overexpression stimulated cell stemness by activating glycolytic pathway. The stimulation of LUAD cell stemness by NCAPG overexpression through the glycolysis pathway was rescued by the addition of 2‐DG. These findings generate novel insights into the role of NCAPG in LUAD progression.

## 2 MATERIALS AND METHODS

### 2.1 Bioinformatics

The mRNA expression data of LUAD were downloaded from TCGA database. Differentially expressed mRNAs (DEmRNAs) were obtained by edgeR differential analysis, and the target gene was identified by combining bioinformatics data and relevant literature. Gene set enrichment analysis (GSEA) of the target gene was done, and correlation analysis was conducted on cell stemness index mRNAsi and glycolysis‐related genes.

### 2.2 Cell culture

Human LUAD cell lines including PC‐9, H1299, and A549 and human bronchial epithelial cell line BEAS‐2B were purchased from BeNa Culture Collection (BNCC, China). Cells were cultured using Roswell Park Memorial Institute‐1640 (Gibco, USA). The medium was supplemented with 10% fetal bovine serum and 100 mg/mL streptomycin plus 100 UI/mL penicillin (Gibco, USA) for incubation at 37°C, 5% CO_2._ ^15^

### 2.3 Cell transfection

sh‐NCAPG, oe‐NCAPG constructed by pcDNA 3.1, and the corresponding negative controls were accessed from RiboBio (China), with 2‐DG and phosphate‐buffered saline (PBS) from Sigma (USA). PC‐9 and A549 cells were seeded into 24‐well plates (5 × 10^4^ cells/well). Transfection was completed by Lipofectamine 3000 (Invitrogen, USA). RT‐qPCR was done to assess transfection efficiency. At 48 h after culture, cells were subjected to functional assays.^15^

### 2.4 qRT‐PCR

Trizol (Invitrogen, USA) was added with DNase I to obtain RNA, which was reverse transcribed to cDNA by SuperScript III® (Invitrogen, USA). The generated cDNA was amplified using qRT‐PCR with reference to the TaqMan method and the Bio‐Rad CFX96 Sequence Detection System (Bio‐Rad, USA). β‐Actin was the internal reference gene. qRT‐PCR results were analyzed. Primers are listed in Table 1.

**TABLE 1:** Primers used in qRT‐PCR.

Gene		Sequence
NCAPG	Forward primer	5′‐ATCCAGAAGTTAGACGGGCAG‐3′
	Reverse primer	5′‐GTGCGCCCTACAATTTTTGGC‐3′
β‐Actin	Forward primer	5′‐CACGAAACTACCTTCAACTCC‐3′
	Reverse primer	5′‐CATACTCCTGCTTGCTGATC‐3′

### 2.5 CCK‐8

Cell viability was tested by the CCK‐8 kit (DOJINDO, Japan). Briefly, cells were seeded into six‐well plates, and the optical density (OD) values at 450 nm were measured at 24, 48, and 72 h using a microplate reader after the addition of 10 μL CCK‐8 diluent for 2 h, separately.^15^

### 2.6 Stem cell sphere‐forming assay

The same number of cells (1000) were seeded into ultra‐low attachment culture dishes and cultured in RMPI‐1640 supplemented with 2% B27 (Thermo Fisher, USA), 20 ng/mL epidermal growth factor, and 10 ng/mL basic fibroblast growth factor (BD, USA) in 10% FBS for 10 days. Cell balls were observed with an inverted microscope and photographed, and the longest diameter was calculated by the scale size in the images using Image‐Pro Plus software.^16^

### 2.7 Western blot

Harvested cells were lysed by RIPA buffer. After sonication, samples were centrifuged at 12 000 *g* for 15 min at 4°C. Total protein concentration was determined by applying DC Protein Assay Kit I (Bio‐Rad, USA). Proteins were transferred to Hybond nitrocellulose membranes (USA) after separation on 12% SDS‐PAGE. 5% skim milk was added to seal the membrane in Tris‐buffered saline (pH 7.5) at room temperature. The membrane was incubated with the primary antibody overnight at 4°C, followed by 4‐h incubation with the secondary antibody at room temperature. Protein bands were developed by ECL kit (Millipore, USA). Protein levels were assessed by ImageJ (USA). Primary antibodies including anti‐CD133, anti‐CD44, anti‐Oct‐4, anti‐HK2, anti‐PKM2, anti‐LDHA, anti‐β‐actin, and secondary antibody anti‐IgG were purchased from Abcam (UK).^16^

### 2.8 Oxygen consumption rate (OCR) and extracellular acidification rate (ECAR)

Metabolic state of cells was measured by XFe96 Analyzer (Seahorse, USA). The day before the experiment, sensor cassette was supplemented with distilled water followed by incubation overnight in a CO_2_‐free incubator at 37°C. Cells were grown overnight on Seahorse XF96 plates (5 × 10^4^ cells/well). On the experimental day, distilled water was changed to XF calibration solution from the sensor cassette, and the media was replaced with Assay Media in the Seahorse XF96 plate. Sensor cassettes and Seahorse XF96 plates were incubated in a non‐CO_2_ incubator at 37°C for 1 h. Following injection of glucose (10 mM), oligomycin (1 μM), and 2‐DG (100 mM), glycolytic capacity was analyzed. Following injection of oligomycin (1 μM), FCCP (1 μM), and rotenone/antimycin A (1 μM), glucose OCR was detected. Both OCR and ECAR were assessed. Background OCR and ECAR that were obtained from wells without cells (only medium) were subtracted automatically on the software.^17^

### 2.9 Detection of pyruvic acid, lactate, citrate, and malate

The production of glycolytic metabolites pyruvic acid, lactate, citrate, and malate in cells was assessed with assay kits. The pyruvate assay kit (Catl. No. A081) and citrate assay kit (Catl. No. A128) were purchased from Nanjing Jiancheng Bioengineering Institute (China). The lactate assay kit (Catl. No. K951) and malate assay kit (Catl. No. K637) were purchased from BioVision (USA).

### 2.10 Statistics

Experiments were replicated three times. Data were expressed as mean ± standard error of mean (SEM). Wilcoxon rank‐sum test was utilized to test the difference between groups. *T*‐tests or one‐way analysis of variance were conducted on GraphPad 8.0 to analyze data from two or more groups. Pearson correlation analysis was performed on correlation of NCAPG with other genes. *P* < 0.05 indicated a significant difference.

## 3 RESULTS

### 3.1 NCAPG expression is high in LUAD tissues and cells

**FIGURE 1:**
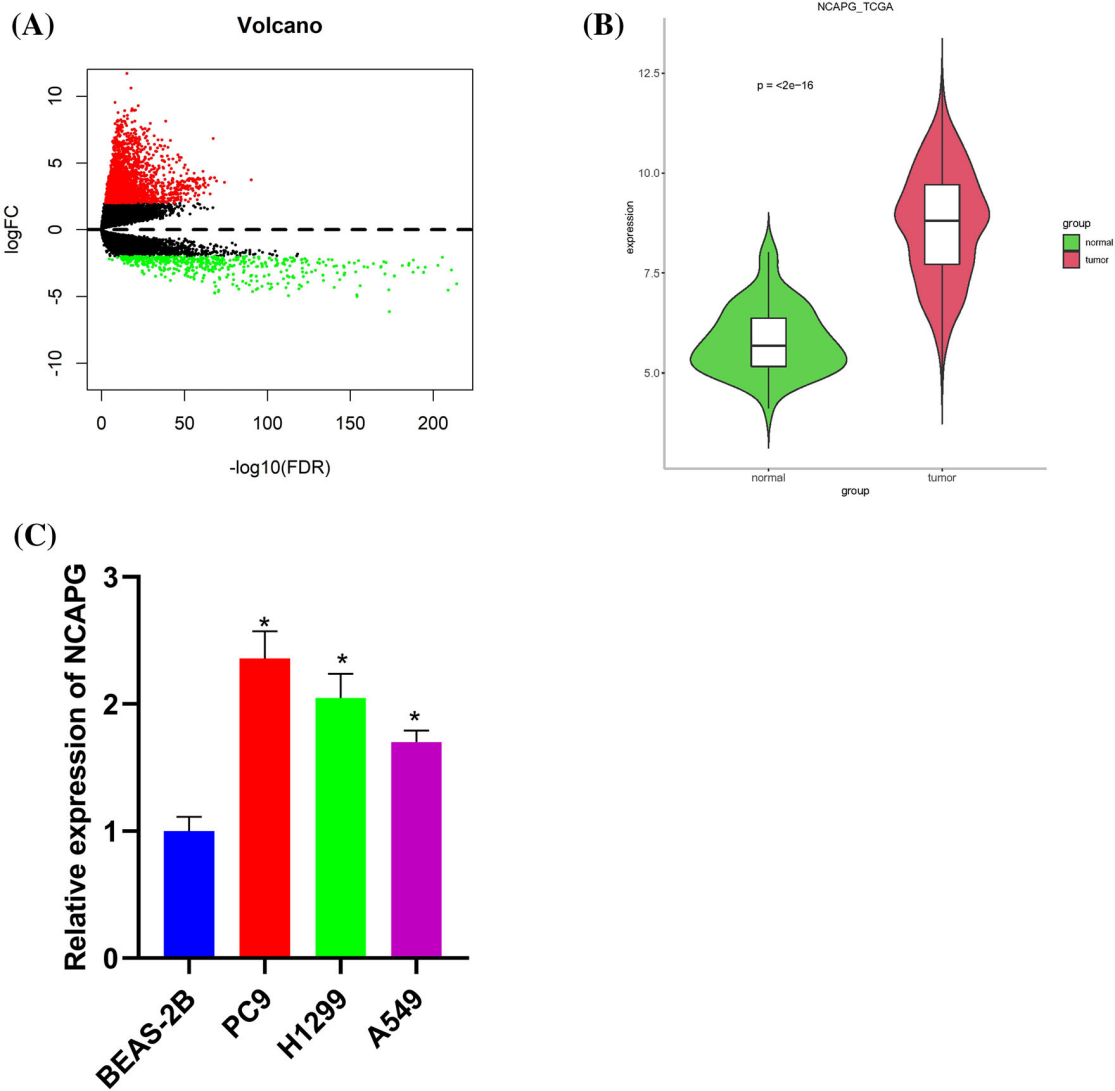
NCAPG expression is high in LUAD tissues and cells. (A) Differentially expressed genes in LUAD tissues and normal tissues from TCGA database. (B) NCAPG expression in LUAD tissues and normal tissues from TCGA database. (C) NCAPG expression in human bronchial epithelial cells (BEAS‐2B) and human LUAD cells (PC‐9, H1299, A549); *P < 0.05.

To unveil the oncogenic role of NCAPG in LUAD patients, firstly, we analyzed the differentially expressed genes in the TCGA data (Figure 1A). Differential analysis of LUAD tissues and normal lung tissues from TCGA data was done using edgeR package. NCAPG was substantially upregulated in LUAD tissues (Figure 1B). NCAPG was notably upregulated in LUAD cell lines PC‐9, H1299, and A549 but not in normal bronchial epithelial cell line BEAS‐2B as revealed by qRT‐PCR (Figure 1C). To sum up, NCAPG was markedly upregulated in LUAD tissues and cells.

### 3.2 NCAPG facilitates LUAD cell stemness

**FIGURE 2:**
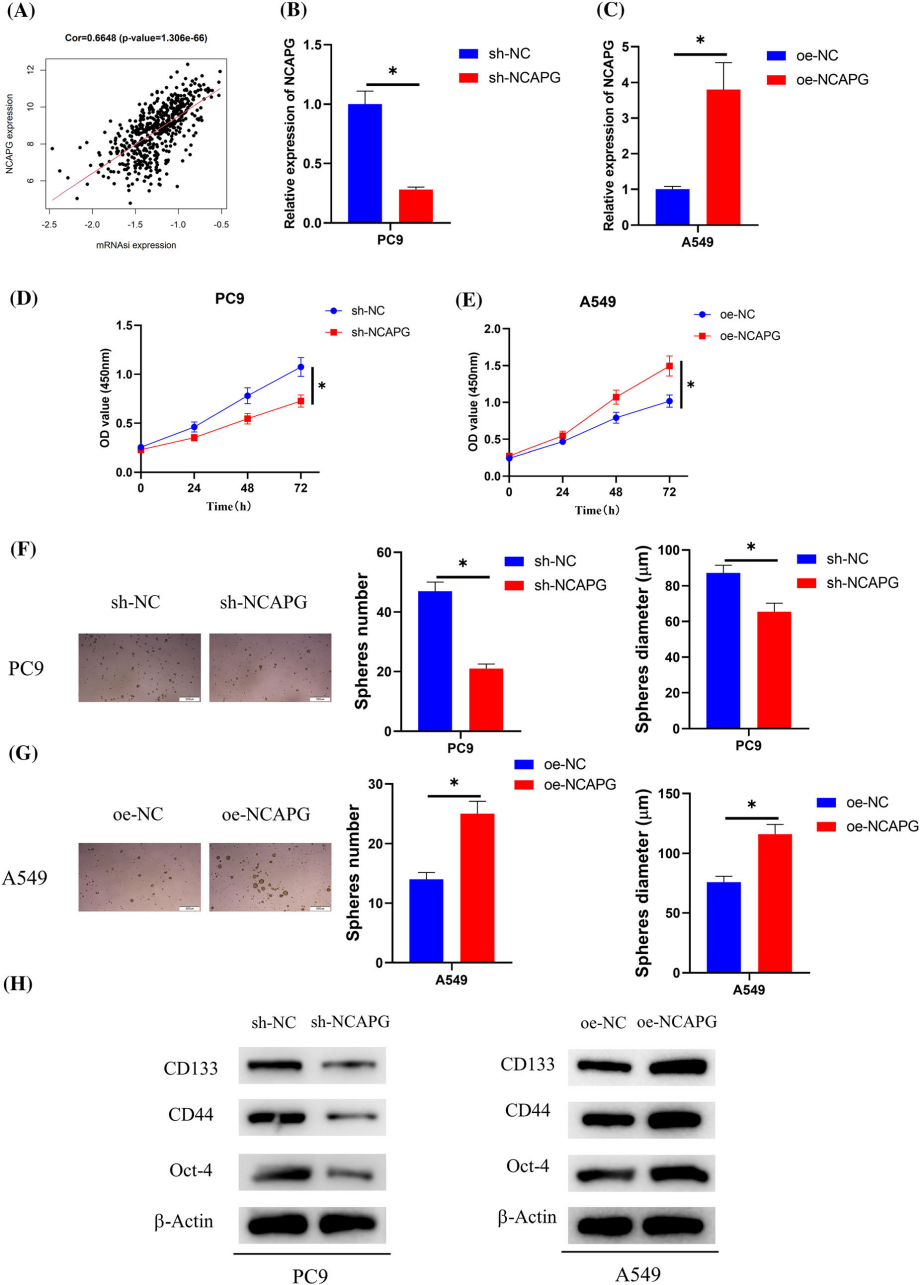
NCAPG facilitates LUAD cell stemness. (A) Pearson correlation analysis of NCAPG and stemness index mRNAsi. (B,C) qRT‐PCR assayed transfection efficiency. (D,E) CCK‐8 assayed cell viability after transfection. (F,G) Cell sphere‐forming assay measured cell sphere‐forming ability after transfection. (H) Western blot assessed protein levels of stem cell markers (CD133, CD44, and Oct‐4) after transfection; *P < 0.05.

NCAPG has been identified as a possible gene related to CSC characterization by transcriptome analysis.^18^, ^19^ The relationship of NCAPG expression with the stemness index of LUAD was therefore investigated, with the results shown in Figure 2A. LUAD stemness index mRNAsi was positively correlated with NCAPG expression in TCGA‐LUAD patients. To investigate the possible role of NCAPG on LUAD cell stemness, sh‐NCAPG was transfected into PC‐9 cells (relatively high NCAPG expression) and oe‐NCAPG into A549 cells (relatively low NCAPG expression). qRT‐PCR assayed transfection efficiency, and results presented that NCAPG levels were reduced in the PC‐9 cell line with NCAPG knockdown (Figure 2B). But NCAPG levels were increased in the oe‐NCAPG cell line (Figure 2C). The results suggested good transfection efficiency, and these two cell lines were allowed for subsequent experiments. For cell viability and stemness tests, CCK‐8 and cell‐forming sphere assays unraveled that sh‐NCAPG was able to reduce the viability and stemness of PC‐9 cells (Figure 2D,F), but oe‐NCAPG remarkably increased the viability and stemness of A549 cells (Figure 2E,G). Western blot unveiled that sh‐NCAPG prominently decreased levels of CD133, CD44, and Oct‐4 in PC‐9 cells, and oe‐NCAPG noticeably elevated levels of these stem cell markers in A549 cells (Figure 2H). Taken together, the results confirmed that NCAPG stimulated LUAD cell stemness.

### 3.3 NCAPG fosters glycolysis in LUAD cells

**FIGURE 3:**
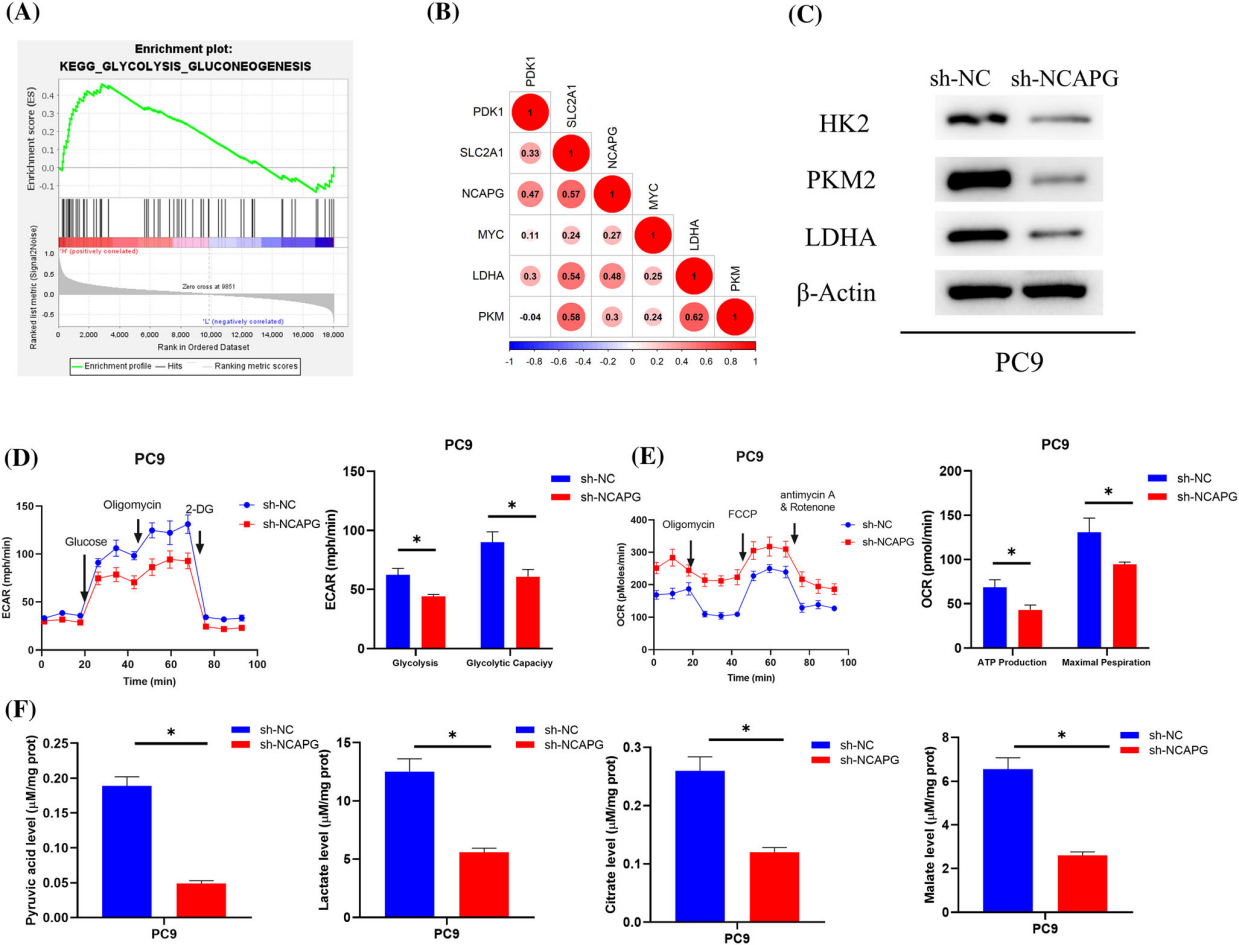
NCAPG fosters glycolysis in LUAD cells. (A) High NCAPG expression was notably associated with glycolysis/gluconeogenesis pathway as revealed through GSEA. (B) Heat map of NCAPG and glycolysis‐related genes via Pearson correlation analysis. (C) Glycolysis metabolism pathway‐related proteins (HK2, PKM2, LDHA) expression after transfection as tested by western blot. (D,E) Seahorse XP 96 analysis of ECAR and OCR in LUAD cells in varying treatment groups. (F) Detection of pyruvic acid, lactate, citrate, and malate in LUAD cells in each treatment group; *P < 0.05.

To investigate the signaling pathway of NCAPG affecting LUAD stemness, GSEA was conducted. High NCAPG expression was noticeably associated with glycolysis/gluconeogenesis metabolism pathway (Figure 3A). Aerobic glycolysis is a hallmark of cancer, including LUAD.^20^ Next, correlation of NCAPG with glycolytic marker genes PDK1, LDHA, PKM, MYC, and SLC2A1 was validated. NCAPG was positively correlated with glycolytic marker genes (Figure 3B). To validate influence of abnormal NCAPG expression on aerobic glycolysis, western blot tested expression of glycolytic metabolic pathway‐related proteins (HK2, PKM2, LDHA) in PC‐9 cells. Their levels were hampered by sh‐NCAPG in PC‐9 cells (Figure 3C). To unravel relationship of NCAPG with metabolism, Seahorse assay was applied to identify metabolic alterations in PC‐9 cells with NCAPG knockdown. OCR and ECAR results illustrated that glycolytic level and glycolytic capacity of LUAD cells were notably reduced in PC‐9 cells with NCAPG knockdown (Figure 3D). The basal and maximal OCRs of LUAD cells were remarkably increased (Figure 3E). The impact of sh‐NCAPG on glycolytic metabolism level in LUAD cells was investigated by detecting Glycolysis/Gluconeogenesis products. sh‐NCAPG rather than sh‐NC markedly hindered production of pyruvic acid, lactate, citrate, and malate (Figure 3F). Together, these findings suggested that NCAPG facilitated aerobic glycolysis in LUAD cells.

### 3.4 NCAPG stimulates LUAD cell stemness through activation of glycolysis pathway

**FIGURE 4:**
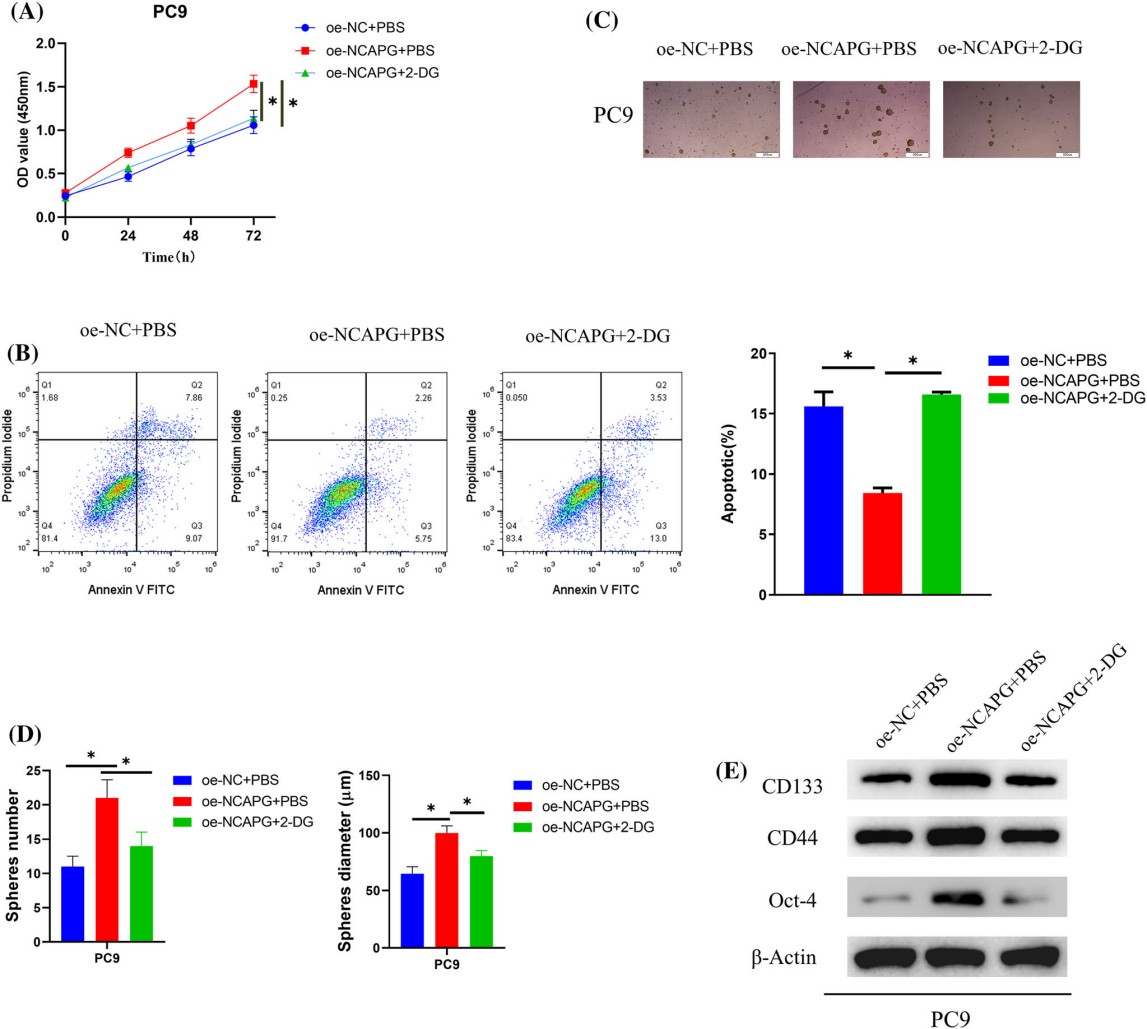
NCAPG stimulates LUAD cell stemness through activation of glycolysis pathway. (A) CCK‐8 assay for cell viability after transfection. (B) Flow cytometry for apoptosis after transfection. (C,D) Cell sphere‐forming assay for cell sphere‐forming ability after transfection. (E) Western blot for expression of stem cell markers (CD133, CD44, and Oct‐4) after transfection; *P < 0.05.

The relationship of NCAPG with LUAD cell stemness via glycolysis was assayed by designing groupings in PC‐9 cells: oe‐NC + PBS, oe‐NCAPG + PBS, and oe‐NCAPG + 2‐DG (glycolysis inhibitor). CCK‐8, flow cytometry, and cell sphere formation assays manifested that NCAPG overexpression substantially elevated viability and stemness of PC‐9 cells and reduced apoptosis, which could be reversed by concurrent 2‐DG treatment (Figure 4A–D). Western blot assessed expression of stem cell markers (CD133, CD44, and Oct‐4). NCAPG overexpression was able to foster levels of these markers, but this stimulative impact was rescued by concomitant 2‐DG treatment (Figure 4E). In summary, NCAPG facilitated LUAD cell stemness by activating glycolysis pathway.

## 4 DISCUSSION

LUAD is a predominant histology of lung cancer and one of the deadliest cancers worldwide.^17^ There is growing evidence that tumor stem cells are involved in tumorigenesis, metastasis, recurrence, and resistance to chemotherapy and radiotherapy, which is one of the reasons tumors are incurable.^1^ The condensin complex gene NCAPG, a cell cycle‐associated condensin, is highly expressed in multiple cancers and facilitates tumor progression.^11^, ^21^ Shi et al^21^ reported that NCAPG fosters proliferation, migration, and invasion of colorectal cancer cells through the Wnt/β‐catenin signaling pathway by binding to β‐catenin. Zhang et al^22^ manifested that NCAPG overexpression leads to aberrant activation of the PI3K/AKT signaling pathway, fostering pancreatic adenocarcinoma cell proliferation. In the present study, the findings confirmed that NCAPG was highly expressed in LUAD tissues and cells. Previously, transcriptome analysis identified NCAPG as a possible key gene implicated in cancer cell stemness.^18^, ^19^ NCAPG was positively correlated with the stemness index as presented by bioinformatics analysis. Cell function experiments unraveled that NCAPG substantially fostered cell stemness, indicating the involvement of NCAPG in modulating LUAD cell stemness. These findings contributed to constraining LUAD cell stemness by targeting NCAPG.

To investigate the relationship of NCAPG with LUAD cell stemness, enrichment analysis results presented that NCAPG was enriched in LUAD glycolysis signaling pathway. NCAPG could activate LUAD cell glycolysis pathway as revealed by cell function assays. Reports on the association of NCAPG with glycolysis pathway in LUAD are less, and thus understanding the relationship is paramount for LUAD glycolysis repression. Cancer cells exhibit metabolic reprogramming characterized by aerobic glycolysis, which is also essential for maintaining cancer stemness.^23^ Bi et al^24^ disclosed that deletion of HDAC11 increases LKB1 transcription by fostering histone acetylation in its promoter region, thus activating the AMPK signaling pathway and hindering the glycolysis pathway, which in turn suppresses cancer stemness and hepatocellular carcinoma progression. Liu et al^25^ reported that isomuciferin constrains the stem cell‐like cell stemness of lung cancer by blocking the MnSOD signaling pathway and hampering glycolysis. Zhu et al^23^ presented that ETV4 facilitates breast cancer stemness via enhancing glycolytic activity. In this study, knockdown of NCAPG represses LUAD cell stemness through hampering glycolysis pathway by rescue experiments.

To our knowledge, this study revealed for the first time the relationship of NCAPG with cell stemness in LUAD and demonstrated that NCAPG fostered LUAD cell stemness by activating glycolysis pathway, supporting the evidence for NCAPG as a possible biomarker in lung cancer. But this study lacked animal and clinical validations of the influences of NCAPG on LUAD cell stemness. More clinical samples need to be collected for the investigation of the mechanism of NCAPG affecting LUAD stemness, laying a theoretical basis for LUAD therapy. In conclusion, this study bolsters the understanding of molecular mechanisms of LUAD development, and these findings suggest that NCAPG may be a new target for LUAD treatment.

## AUTHOR CONTRIBUTIONS

Zuwang Zhang is responsible for conception, design, and provision of study materials, Dongdong Qi is responsible for collection and assembly of data, Xun Liu is responsible for data analysis and interpretation, and Poming Kang is responsible for manuscript writing, conception, and design. All authors approved the final version.

## CONFLICT OF INTEREST STATEMENT

The authors report no conflict of interest.

## ETHICS STATEMENT

No animal/human cell used.

## Data Availability

The datasets used and/or analyzed during the current study are available from the corresponding author on reasonable request.
